# Sustained Delivery of a Monoclonal Antibody against SARS-CoV-2 by Microencapsulated Cells: A Proof-of-Concept Study

**DOI:** 10.3390/pharmaceutics14102042

**Published:** 2022-09-24

**Authors:** Assem Ashimova, Askhat Myngbay, Sergey Yegorov, Baurzhan Negmetzhanov, Irina Kadyrova, Angelina Yershova, Ulpan Kart, Matthew S. Miller, Gonzalo Hortelano

**Affiliations:** 1 School of Sciences and Humanities, Nazarbayev University, 53 Kabanbay Batyr Ave, Nur-Sultan 010000, Kazakhstan; 2 Centre for Life Sciences, National Laboratory Astana, Nazarbayev University, 53 Kabanbay Batyr Ave, Nur-Sultan 010000, Kazakhstan; 3 Michael G. DeGroote Institute for Infectious Disease Research, McMaster Immunology Research Centre, Department of Biochemistry and Biomedical Sciences, McMaster University, Hamilton, ON L8S 4L8, Canada; 4 Research Centre, Karaganda Medical University, 40 Gogol St, Karaganda 100008, Kazakhstan; 5 Faculty of Biological Sciences, Friedrich-Shiller-University Jena, Fürstengraben 1, 07743 Jena, Germany

**Keywords:** COVID-19, SARS-CoV-2, IgG, monoclonal antibody, CR3022, cell encapsulation

## Abstract

Background: Monoclonal antibody (mAb) therapy is a promising antiviral intervention for Coronovirus disease (COVID-19) with a potential for both treatment and prophylaxis. However, a major barrier to implementing mAb therapies in clinical practice is the intricate nature of mAb preparation and delivery. Therefore, here, in a pre-clinical model, we explored the possibility of severe acute respiratory syndrome coronavirus 2 (SARS-CoV-2) mAb delivery using a mAb-expressing encapsulated cell system. Methods: Murine G-8 myoblasts were transfected with plasmids coding for the heavy and light chains of CR3022, a well-characterized SARS-CoV-2 mAb that targets the Spike receptor binding domain (RBD), and then encapsulated into alginate microcapsules. The microcapsules were then intraperitoneally implanted into immunocompetent (C57/BL6J) mice and changes in circulating CR3022 titres were assessed. The in vitro and ex vivo characterization of the mAb was performed using western blotting, RBD ELISA, and microscopy. Results: Transfected G-8 myoblasts expressed intact CR3022 IgG at levels comparable to transfected HEK-293 cells. Cell encapsulation yielded microcapsules harbouring approximately 1000 cells/capsule and sustainably secreting CR3022 mAb. Subsequent peritoneal G-8 microcapsule implantation into mice resulted in a gradual increase of CR3022 concentration in blood, which by day 7 peaked at 1923 [1656–2190] ng/mL and then gradually decreased ~4-fold by day 40 post-implantation. Concurrently, we detected an increase in mouse anti-CR3022 IgG titers, while microcapsules recovered by day 40 post-implantation showed a reduced per-microcapsule mAb production. Summary: We demonstrate here that cell microencapsulation is a viable approach to systemic delivery of intact SARS-CoV-2 mAb, with potential therapeutic applications that warrant further exploration.

## 1. Introduction

Although coronavirus disease (COVID-19) is still a serious global health concern, the rapid development of preventive and therapeutic antiviral modalities triggered by the global initiative to end the pandemic has resulted in a reduction of global COVID-19 infection and mortality [1]. The list of therapeutics recommended for use is growing and now includes anti-inflammatory corticosteroids, IL-6 receptor blockers (tocilizumab or sarilumab), the Janus kinase inhibitor baricitinib, and the severe acute respiratory syndrome coronavirus (SARS-CoV-2)-neutralizing monoclonal antibodies (sotrovimab, casirivimab and imdevimab) [2]. Of these, SARS-CoV-2-targeting monoclonal antibodies (mAbs) are unique due to their high specificity of action, namely, neutralizing SARS-CoV-2 [3], and, in support of this, emerging clinical trial evidence indicates that mAbs can reduce SARS-CoV-2 viral loads and improve clinical outcomes in specific patient sub-categories. In addition, some studies have suggested that SARS-CoV-2 mAbs could be used prophylactically in COVID-19 exposed subjects [3].

However, a major barrier to implementing mAb therapy in clinical practice is the complex and expensive nature of mAb manufacturing, whereby mAb production and purification is notoriously costly and presents a challenge to product development and pre-clinical modification [3]. Moreover, parenteral mAb delivery also presents clinical challenges, and although mAb therapy has overall low reactogenicity, patients do experience mild-to-moderate injection site and infusion-related reactions [3].

Here, we hypothesized that SARS-CoV-2 mAbs could be effectively delivered by mAb-expressing allogeneic cells, encapsulated to enhance cell viability and protection from host immune responses [4,5]. Delivery of therapeutic agents by microencapsulated single cell populations has been successfully used in both animal models and humans to treat various conditions, including diabetes mellitus [6,7], anemia [8], cancer [9], and neurodegenerative disease [10,11]. Thus, we rationalized that SARS-CoV-2 mAb delivery by encapsulated cells could be an effective alternative to bolus mAb injection-based COVID-19 treatment. Therefore, we tested this hypothesis in a pre-clinical model and demonstrate that implanted microcapsules in vivo can deliver systemically detectable titres of a SARS-CoV-2 mAb sustained up to day 40 post-implantation.

## 2. Methods

### 2.1. Cells

G-8 murine myoblasts (CRL-1456) and HEK-293 human embryonic kidney cells (CRL-1573) were purchased from American Type Culture Collection (ATCC, Manassas, VA, USA) and cultured in Dulbecco’s Modified Eagle Medium (DMEM, high glucose, GlutaMAX) (#61965-026, Gibco, Waltham, MA, USA) supplemented with 10% fetal bovine serum (FBS) (#26140-079, Gibco) and 1% penicillin/streptomycin (#15140-122, Gibco) at 37 °C and 5% CO_2_.

### 2.2. mAb Expression

The CR3022 plasmid set (NR-53260), encoding the vH and vL sequences subcloned into mammalian expression vectors and fused to the C-terminal constant regions of human IgG1 (hIgG1) heavy or human Ig kappa (hIgκ) light chains, respectively, was sourced from BEI Resources (NIAID, NIH, Bethesda, MD, USA). G-8 and HEK-293 cells were co-transfected with the CR3022 plasmids at 60% confluence in 100 mm cell culture dishes using liposome forming compound Escort-III (L3037, Sigma, St Louis, MO, USA) following the manufacturer’s protocol. Briefly, a pre-incubated mixture of 5 μg of each plasmid and 5 μL of Escort-III in 990 μL of Opti-MEM (#31985-070, Gibco) was incubated with the cells for 6 h at 37 °C. Cells stably expressing the transgene were then selected by supplementing cell culture with 400 μg/mL of zeocin (R25005, Invitrogen, Carlsbad, CA, USA) and 10 μg/mL of blasticidin (#15205, Sigma-Aldrich) for 2 weeks. mAb production was assessed by western blotting and ELISA.

### 2.3. Western Blotting

Western blotting was used to validate the secreted mAb size and specificity. Spike (NR-722, BEI Resources, NIAID, NIH, Bethesda, MD, USA) and nucleocapsid (NR-48761, BEI Resources) proteins were separated on a 10% SDS–PAGE gel and transferred onto a PVDF (Polyvinylidene fluoride) membrane (#116-2807, Bio-Rad, Munich, Germany) for 1 h at 350 mA. The membrane was then blocked with 5% (*w*/*v*) dry milk powder (#B501-0500, Rockland, NY, USA) in PBST (Phosphate Buffered-Tween) (1 × PBS, 0.05% Tween 20) at RT (Room temperature) for 1 h, washed thrice in TBST and then blotted with cell culture supernatant at 4 °C overnight. mAb detection was done using HRP-conjugated anti-human abs specific to either intact human IgG (rabbit ab6759, Abcam, Cambridge, UK) or the IgG Fc region (goat ab97225, Abcam) at 1:10,000 dilution and incubated on the membrane for 1 h at RT. To assess the mAb in a denatured state, purified (on Protein G HP Spin Trap, #28903134, Cytiva, Malborough, MA, USA) cell supernatant was heated for 5 min at 95 °C at reducing conditions prior to SDS–PAGE separation. The BEI human monoclonal antibody CR3022 (NR-52481, BEI Resources) at 500 ng/well (~20 ng/μL) was used as a reference and molecular weight was estimated using the PageRuler Prestained Protein Ladder (#26616, Thermo Fisher Scientific, Waltham, MA, USA). Imaging was performed on the ChemiDoc MP Imaging System (Bio-Rad).

### 2.4. SARS-CoV-2 ELISA

A total of 96-well plates (#460984, Flat-Bottom Immuno Nonsterile, Thermo Fisher Scientific) were coated overnight at 4 °C with 2 ng/μL of RBD (NR-72946, BEI Resources). The next day, wells were washed thrice with PBST, followed by blocking with 5% milk in PBST for 1 h at RT and subsequent PBST wash. Cell culture supernatant or plasma (at 1:100 dilution, 100 μL) was added to each well and incubated for 2 h at RT. After washing, plate was incubated for 1 h with 1:100,000 diluted horseradish peroxidase (HRP)-conjugated goat anti-human IgG (Fc-specific, ab97225, Abcam). After a final wash, TMB (3,3′,5,5′-tetramethylbenzidine) chromogenic substrate (#34029, Thermo Fisher Scientific) was added and incubated for 15 min in the dark. The reaction was stopped using 50 μL of stop solution (#SS04, Thermo Fisher Scientific). Total human IgG detection was done following the RBD detection protocol outline above, with the following exceptions: plates were coated with primary goat anti-human IgG (Fc specific, Sigma-Aldrich, I2136) at 1/10,000 dilution, while rabbit anti-human IgG H+L(ab6957) mAb at 1:100,000 was used as a secondary mAb.

Absorbance was measured at 450 nm on the Varioscan Flash microplate reader (Thermo Fisher Scientific). Standard curves were constructed by diluting down the CR3022 (NR-52481) mAb in PBS starting at a maximum concentration of 4 µg/mL. The mAb concentrations were calculated using the standard curves and using experimental optical density (OD) readouts normalized to average baseline values obtained for respective controls, i.e., untransfected supernatant or blood plasma from CR3022-naïve G-8 capsule implanted animals by subtracting the respective control OD values from the experimental OD readouts.

Validation of the in-house ELISA was performed against a commercially available S-IgG assay (Euroimmun Medizinische Labordiagnostika AG, Lübeck, Germany) on human plasma obtained from COVID-19 seroprevalence and vaccination studies approved by the Research Ethics Board of the Karaganda Medical University on 6 April 2020 (Ethics Protocol #45) [12,13]. Out of the 20 human samples, 10 were obtained from vaccinated (SARS-CoV-2 Ab+) subjects and 10 were pre-pandemic (SARS-CoV-2 Ab-negative) samples randomly chosen based on availability.

### 2.5. Cell Encapsulation

Alginate solution (1.5%) was prepared by dissolving low viscosity alginate powder (Keltone LV, 50–80 kD MW#9005-38-3, San Diego, CA, USA) in 0.9% sodium chloride (#7647-14-5, Fisher Scientific, UK) overnight at 37 °C and filtered using a 0.22 μm filter (#99722, TPP). G-8 myoblasts were suspended in alginate solution at a concentration of 5 × 10^6^ cells/mL. Alginate microbeads were generated using an electrostatic encapsulator Var1 (Nisco Engineering Inc., Zurich, Switzerland) as described earlier [10,11,12] with minor adjustments. Briefly, cell suspension was pumped through a NISCO Var-1 encapsulator (Nisco Engineering Inc., Zurich, Switzerland) (voltage = 7.10 kV; flow rate = 20 mL/h) into a vial containing cold 100 mM CaCl_2_ solution, yielding microcapsules of 300–500 µm in diameter. Cell-loaded beads were then washed with saline solution and cross-linked with poly-l-lysine (PLL, 29 kDa, P7890, Sigma) for 6 min and with an outer layer of alginate for 4 min. Finally, DMEM was added and the solution containing the beads was moved to an incubator. Viability of microencapsulated cells was assessed using the trypan blue exclusion assay, whereby 20 μL of microcapsules and 20 μL of trypan blue (Invitrogen, USA) were placed on a microscope slide and crushed by a glass coverslip to release the encapsulated cells, prior to visualization and manual quantification on an inverted light microscope (Ceti, Medline Scientific, Chalgrove, UK) or using an automated cell counter Countess 3 (Thermo Fisher). Electron microscopy-aided imaging was performed using a JSM-IT200LA (JEOL, Akishima, Japan) scanning electron microscope on microcapsules air-dried for 1 h at RT.

### 2.6. Animal Studies

All animal procedures were conducted in accordance with the Animal Ethics Guidelines of Nazarbayev University and approved by the National Center for Biotechnology (Nur-Sultan, Kazakhstan) on 3 May 2021 (Ethics Protocol #5). Eight-week-old C57BL/6J female mice were purchased from the animal facility of the National Center for Biotechnology, where the animals (originally sourced from Charles River, Germany) were maintained in a specific pathogen-free environment. There were three experimental groups: group #1 was implanted with microcapsules harbouring non-transfected cells (*n* = 4), group #2 was implanted with microcapsules harbouring transfected cells (*n* = 6), and group #3 was injected intraperitoneally with 1 mL of 5 mg/mL polyclonal human IgG (#A50170H, Meridian Life Sciences, Memphis, TN, USA) in PBS solution using an 18G catheter (*n* = 2). Prior to capsule implantation, animals were anaesthetised with isoflurane (Harvard Apparatus, Cambridge, UK). The microcapsule concentration in the stock microcapsule solution was quantified by manual counting using light microscopy. Microcapsules were implanted intraperitoneally using a G18 catheter containing 3 mL of microcapsule solution at an average ~5000 microcapsules per 1 mL. Plasma was isolated from tail vein blood by centrifugation at 2000× *g* for 10 min and stored at −20 °C before use. Animals were sacrificed 40 days after implantation by anesthesia overdose and microcapsules were retrieved (available volume ~2.5 mL) and analyzed by inverted light microscopy (EVOS FL Auto, Thermo Fisher Scientific). The retrieved microcapsules were washed in PBS and analyzed for cell viability and mAb secretion.

### 2.7. Statistical Analysis

All analyses were performed in GraphPad Prism V.9.3.1 software (GraphPad Software, San Diego, CA, USA). We used unpaired *t*-test to compare differences between groups, and 95% confidence intervals (CI) were calculated using the binomial “exact” method.

### 2.8. Role of the Funding Source

The funder of the study had no role in study design, data collection, data analysis, data interpretation, or writing of the report.

## 3. Results

### 3.1. G-8 Myoblasts Are Capable of Sustained CR3022 Expression

**Figure 1:**
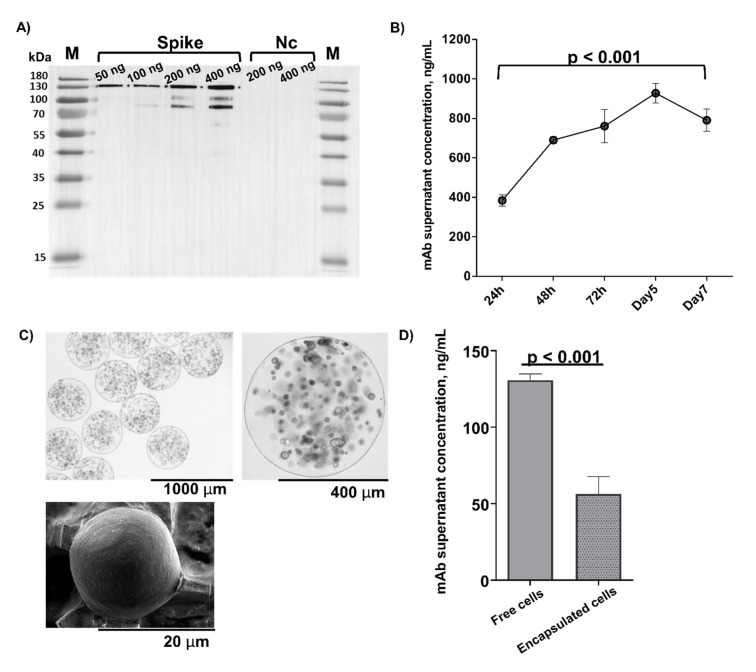
SARS-CoV-2 monoclonal antibody production in cells transfected with the CR3022 heavy and light chains. For western blot analysis (**A**) different amounts of Spike (50–400 ng) and nucleocapsid (Nc, 200–400 ng) proteins were separated on a 10% SDS–PAGE gel, transferred onto a PVDF membrane and then blotted with G-8 cell culture supernatant; probing was done using an HRP-conjugated rabbit IgG specific to both the heavy and light chains of human IgG. Lanes M: protein ladder; Spike lanes: S protein at 50, 100, 200, and 400 ng. Lanes N: N protein at 200 and 400 ng. kDA = kilodaltons. (**B**) CR3022 expression in cultured G-8 cells quantified using receptor binding domain (RBD) ELISA and normalized to the untransfected supernatant readout. Cell media were changed every 24 h. Lines and brackets represent the geometric means and 95% confidence intervals, respectively. (**C**) Microphotographs of G-8 cell-containing alginate microcapsules obtained using light (top left panel at ×4 and top right panel at ×10 magnification) and electron microscopy (bottom left panel at ×800 magnification). (**D**) Supernatants collected from free and encapsulated G-8 cells stably expressing CR3022 at 48 h after last media change. Supernatants (at 1/100 dilution) were incubated on RBD-coated ELISA plates. Lines and brackets represent the geometric means and 95% confidence intervals, respectively. In (**B**,**D**): mAb concentrations were calculated using a standard curve generated using a CR3022 mAb (NR-52481) from BEI on experimental readouts normalized to the corresponding baseline control (untransfected supernatant) OD readouts (see Methods).

Since the C mAbR3022-expressing plasmids have been validated in HEK-293 cells (BEI, NIH) but not in murine G-8 myoblasts, we first assessed the production of CR3022 by murine G-8 myoblasts. Both western blotting and ELISA assays demonstrated that CR3022 expression by G-8 myoblasts was comparable to that of HEK-293 cells (Figure 1A and Figure S1). The secreted mAb (molecular weight ~150 kDa) was confirmed to bind to Spike, but not to the nucleocapsid protein of SARS-CoV-2 by western blotting (Figure 1A, Figure S1 and Figure S2). Production of mAb by cultured G-8 myoblasts gradually increased post-transfection and was sustained at a level of ~310 ng/10^6^ cells per day as quantified by RBD-ELISA (Figure 1B).

### 3.2. G-8 Microcapsules Secrete Detectable Levels of CR3022

Ideally, cells used for microencapsulation should exhibit low immunogenicity and have a non-tumorigenic profile. Additionally, encapsulated cells should be well-characterized and reproducible, and logistically feasible and ethical to obtain [4]. To a substantial extent, fetal murine myoblast cell line G-8 fulfils these characteristics, in addition to its ability for sustainable transgene expression, making it a preferred cell line for long-term cell encapsulation and microcapsule implantation [14,15,16,17,18].

Hence, we next encapsulated G-8 cells stably expressing CR3022 in alginate microcapsules at a concentration of ~1000 cells/capsule using our previously validated protocol that yields microcapsules harbouring highly viable cells [17,18]. This approach yielded spherical microcapsules averaging ~106 [range = 79–110] μm in diameter and containing pores with an average diameter of 595 [range = 306–920] nm (Figure 1C) and ~95% cell viability (Figure S1). Microencapsulated G-8 cells secreted CR3022 mAb albeit at lower levels compared to free (unencapsulated) cells (Figure 1D), and comparable to the secretion level of encapsulated human HEK293 cells (Figure S1). Overall, cultured G-8 microcapsules sustainably secreted detectable levels of CR3022 mAb into the media supernatant.

### 3.3. In Vivo Microcapsule Implantation Results in Systemically Detectable CR3022 mAb

**Figure 2:**
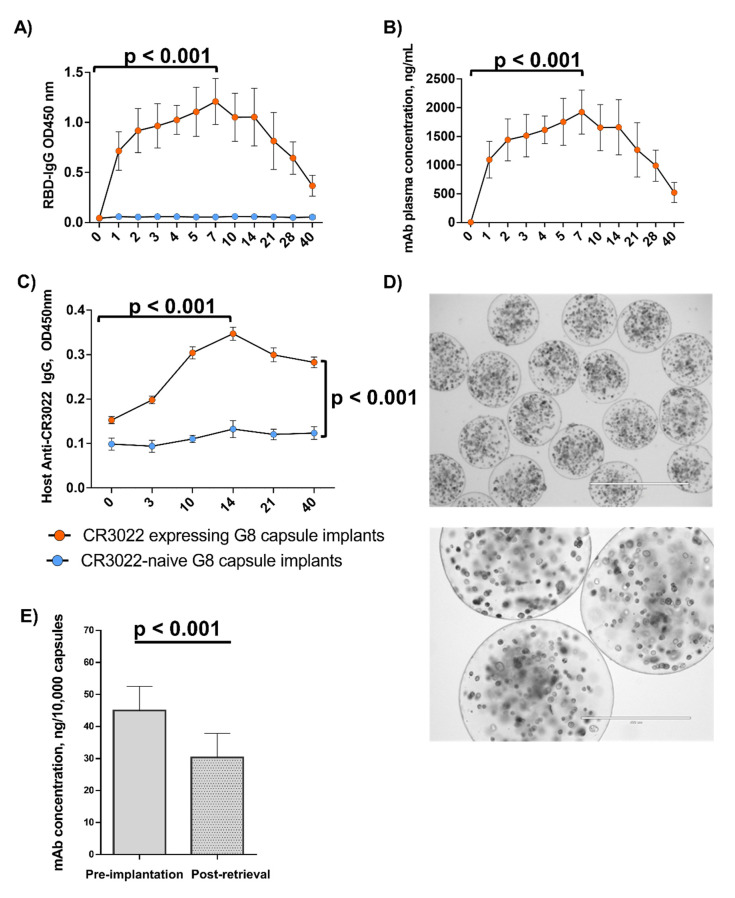
Changes in circulating SARS-CoV-2 mAb titres after microcapsule implantation. Plasma for the ELISA assays was extracted from mouse blood collected pre-implantation (day 0) and on consecutive days up to day 40 post-implantation. (**A**,**B**) RBD-IgG ELISA results depicted as OD450 measurements (**A**) and as mAb concentration normalized to the CR3022-naïve G-8 capsule implanted mouse plasma. (**C**) Mouse anti-human ab response, measured by ELISA. (**D**) Microphotographs of G-8 cell-containing alginate microcapsules extracted from the peritoneal cavity at 40 dpi. Microphotographs were obtained using light microscopy at ×4 (top panel) and ×10 (bottom panel) magnifications. (**E**) CR3022 secretion (defined as RBD-binding mAb concentration per capsule) pre-implantation and post-retrieval at day 40. Lines and brackets represent the geometric means and 95% confidence intervals, respectively. OD, optic density. In (**A**–**C**): The experimental and control groups consisted of six and four mice, respectively. In (**A**,**B**,**E**): mAb concentrations were calculated using a standard curve generated using a CR3022 mAb (NR-52481) from BEI on experimental readouts normalized to the corresponding baseline control (blood plasma from CR3022-naïve G-8 capsule implants) OD readouts (see Methods).

Since the therapeutic value of microcapsules would depend on their capacity to deliver and maintain clinically meaningful mAb levels in vivo, we next implanted the CR3022-expressing encapsulated G-8 cells intraperitoneally in immunocompetent mice (*n* = 6) and monitored CR3022 levels in blood post-implantation using RBD-specific ELISA (Figure 2A,B). CR3022 was detectable in blood plasma starting at 24 hpi and gradually increased in concentration until 7 days post-implantation (dpi) when it reached a peak concentration of 1923.0 [95% CI, 1656.1–2189.9] ng/mL (Figure 2B). Subsequently, CR3022 titers gradually declined, albeit by 40 dpi, and remained detectable at a concentration of 522.0 [95% CI, 400.8–642.7] ng/mL (Figure 2B). A control group of mice received encapsulated untransfected cells (*n* = 4); no circulating mAb was detected in this group. There was a further group of animals injected with polyclonal human IgG at clinical doses (*n* = 4); in this group the RBD-ELISA signal was approximately at the background level, consistent with the specificity of RBD binding by CR3022 but not to polyclonal human IgG (Figure S3), while total human IgG load was higher in the bolus-injected group compared to the microcapsule-implanted animals (Figure S4).

### 3.4. Host Anti-CR3022 Response and Per-Capsule mAb Expression Changes

We next explored whether the observed decline of CR3022 titers post-7dpi was attributable to emergence of host immune response and/or was due to a reduction in per capsule mAb secretion. Therefore, we first assessed changes in anti-CR3022 antibodies in mouse plasma over the period of implantation (Figure 2C). We observed that mouse anti-CR3022 IgG titres gradually increased, peaking at approximately days 10–14 (Figure 2C). We then retrieved implanted microcapsules from the peritoneal cavity at 40 dpi and assessed cell viability and CR3022 production by the retrieved microcapsules in culture. There was no obvious cellular infiltration around the microcapsules within the peritoneal cavity at 40 dpi (Figure 2D). Compared to pre-implantation, at 40 dpi the viability of cells from the retrieved capsules was mildly lower (~85% vs. ~95%), while CR3022 secretion was reduced from 45 to 30 ng per 10^4^ microcapsules as quantified by RBD ELISA (Figure 2E). Overall, CR3022 titers remained detectable over a period of 40 dpi, but waned likely due to both host immune response against the human mAb, and possibly due to a reduced CR3022 production per-microcapsule.

## 4. Discussion

The pressure caused by the COVID-19 pandemic to develop effective therapies has resulted in a rapid advancement of different approaches for disease treatment and prophylaxis. One such approach is based on the use of mAbs and, although very promising, it has been constrained by the challenges associated with mAb production and delivery [3]. Therefore, in this proof-of-concept study, we explored the use of cell microencapsulation [4,5] for sustained delivery of a well-characterized human SARS-CoV-2 mAb, CR3022, in a pre-clinical mouse model. We demonstrate that upon implantation alginate microcapsules containing CR3022-secreting G-8 myoblasts raise blood CR3022 titres, which remain detectable for up to 40 days.

Our current data and earlier experience with the cell encapsulation technology [4,14,15,16,17,18,19] indicate that encapsulated cell-aided mAb delivery is amenable to optimization for different indications, such as COVID-19 treatment versus prophylaxis, by adjusting per-capsule mAb expression. Thus, although peak CR3022 titres in our in vivo studies were ~100 fold lower than in recent clinical trials of bolus casirivimab and imdevimab infusion [20,21,22], we believe that by increasing the quantity of implanted microcapsules and/or by enhancing per-cell mAb expression it would be possible to attain clinically meaningful systemic mAb titres. Overall, however the mAb titres seen in our study are similar to other reports of intravenous mAb administration in humans [23] and reports from mice, where microencapsulated hybridoma cells delivered an average of 0.9 ug/mL IgM in plasma [24].

Cell encapsulation-aided mAb delivery is particularly advantageous compared to bolus mAb administration in contexts requiring a gradual mAb release sustained over a long period. This would be the case of a prophylactic mAb administration, where the aim would be to maintain neutralizing mAb titres at a level needed to prevent or reduce SARS-CoV-2 infection. Since microcapsules would provide a more gradual mAb delivery, we expect that this would also result in a reduction of reactogenicity typically associated with bolus mAb infusions. Furthermore, given the known shortfalls of monotherapy and higher likelihood of SARS-CoV-2 resistance development associated with monotherapy, microencapsulation of cells secreting different mAbs would allow sustained delivery of combined mAbs with a potentially stronger prophylactic or therapeutic effect. As data emerge on drug-resistant virus strains and mAb efficacy in different populations, microencapsulation could combine microcapsulated cells expressing different mAb sub-types or targeting different epitopes. The microencapsulation system could also be utilized to deliver other mAbs of therapeutic value, such as IL-6 receptor blockers (tocilizumab or sarilumab) and potentially be used in medical conditions other than COVID-19.

Our choice of CR3022 as the SARS-CoV-2 mAb for this proof-of-concept study was dictated by its well-characterized nature and its availability in both protein and plasmid form for research applications. This mAb was originally characterized as a SARS-CoV-1 neutralizing mAb via a large-scale library screen and subsequently shown to neutralize SARS-CoV-2 [25,26]. However, the neutralizing capacity of CR3022 has been shown to differ between assays, and a recent report demonstrated that CR3022 neutralizes SARS-CoV-2 without inhibiting the ACE2-RBD interaction [27]. The recent rapid expansion of data available on clinically relevant SARS-CoV-2 mAbs has provided an unprecedented opportunity to use these mAbs in various combinations in COVID-19 treatment and prophylaxis. Thus, we believe that mAbs other than CR3022 can and should be used in the next steps of microencapsulation-aided mAb delivery.

The level of mAb peaked on day 7 and then gradually diminished until the end of the experiment on day 40. The gradual increase in mAb concentration in blood is evidence of a sustained delivery of mAb from the microcapsules in vivo. Since the microcapsules remained intact and free from overgrowth upon retrieval on day 40, and the viability of the encapsulated cells was sustained, it is tempting to speculate that the observed decline in blood CR3022 was in part contributed by an immune response of the host to CR3022. The half-life of the human mAb CR3022 has been reported to be shorter in mice than in humans [28], and we speculate that a greater accumulation of the mAb over time would be observed if this mAb were expressed in humans. Another factor contributing to the gradual decline of CR3022 titres may have been the reduced per-capsule CR3022 expression, which could have occurred due to plasmid loss by encapsulated cells, as well as due to hypoxia, waste build-up, and declined nutrient supply gradually building up around the microcapsules in the peritoneal cavity [19]. Notably, in line with our earlier work demonstrating a successful microcapsule implantation over a 120 day-period [17], no cell overgrowth was observed around the microcapsules upon retrieval on day 40 post-implantation (see Figure 2D), highlighting the low immunogenicity of microcapsules. The use of a GMP-grade alginate largely depleted of endotoxins would further reduce the immunogenicity of the microcapsules in a clinical setting.

This study was designed as a proof-of-concept to assess the feasibility of using genetically engineered encapsulated cells for the sustained delivery of functional mAb in a pre-clinical animal model without concurrent SARS-CoV-2 exposure or active infection. Here we show that the mAb produced by engineered myoblasts is of the expected size and shows specificity targeting and binding to the intended receptor binding domain (RBD) of the Spike protein. Further, the mAb has limited neutralizing activity, in agreement with the available published literature of the CR3022 mAb [29]. Prior studies showed delivery of mAb from hybridoma cell lines [30]. Such strategy resulted in sustained delivery of therapeutic levels of mAb in various animal models [31]. However, hybridoma cells are murine cells obtained from the fusion of a B lymphocyte and a cancer myeloma cell that cannot be considered for human implantation. The feasibility of using encapsulated genetically engineered cells that are not professional antibody secreting cells, as shown in this study, broadens the therapeutic potential spectrum of encapsulated technology to deliver mAb.

The goal of this study was not to assess the prophylactic or therapeutic effect of encapsulated murine myoblasts. Myoblasts have attractive properties as encapsulated cells, but murine myoblasts would not be considered for human implantations. Additionally, the therapeutic potential of the microcapsule strategy against COVID-19 is directly dependent on the ability of the delivered mAb to effectively bind and block cell entry to SARS-CoV-2. This, in turn is dependent on the sequence of the mAb(s) to be secreted. The main goal of this study was to assess the feasibility of genetically engineered cells to deliver sustained and functional human mAb. The ability of genetically engineered human cells (HEK 293) to deliver functional mAb has also been shown in this study.

This proof-of-concept study did not assess different microcapsule dosing regimens to study dose-dependent pharmacodynamics, nor did it evaluate the long-term fate of the implanted microcapsules. In addition, we did not directly assess the longevity of microcapsules beyond 40 dpi, although the biocompatibility of alginate microcapsules has been extensively studied [30,31]. Alginate has a rate of degradation in vivo that is modulated by the concentration of crosslinking agents [31]. Notably, in this work we delivered microcapsules intraperitoneally, a route of microcapsule delivery that has been standard in the field. However, alternative sites of microcapsule implantation should be compared as part of further optimization of the approach for use in clinical applications. Lastly, a valid concern pertains to potential escape, proliferation, and migration of drug-selected cells from microcapsules in vivo. An additional safety feature to control the longevity of encapsulated cells would be to genetically modify cells to render them sensitive to an antibiotic, which could be administered should it become necessary to reverse the treatment.

Taken together, our results indicate that cell microencapsulation is a feasible approach for systemic delivery of an intact SARS-CoV-2 mAb in a pre-clinical model, warranting its further exploration for use in clinical practice. Future research will focus on optimizing the pharmacodynamics and pharmacokinetics of this approach for specific indications involving COVID-19 prophylaxis and treatment.

## Data Availability

The data presented in this study are available within the article and Supplementary Materials.

## Supplementary Materials

The following supporting information can be downloaded at: https://www.mdpi.com/article/10.3390/pharmaceutics14102042/s1, Figure S1: SARS-CoV-2 antibody detection by Western blotting and ELISA; Figure S2: SARS-CoV-2 monoclonal antibody production in cells transfected with the CR3022 heavy and light chains; Figure S3: SARS-CoV-2 monoclonal antibody detection in mice over 40 days of implantation; Figure S4: Total human IgG detection in mice over 40 days of implantation.
