# Supplementary material for: Sustained Delivery of a Monoclonal Antibody against SARS-CoV-2 by Microencapsulated Cells: A Proof-of-Concept Study
Source: Pharmaceutics. 2022 Sep 24;14(10):2042. doi: 10.3390/pharmaceutics14102042 (PMC9607555; doi:10.3390/pharmaceutics14102042)
Supplement: Supplementary file 1 [file pharmaceutics-14-02042-s001.zip › pharmaceutics-1818338-supplementary.pdf]

Supplementary Materials: Sustained SARS-CoV-2 monoclonal antibody delivery by encapsulated cells: A proof-of-concept study

Assem Ashimova, Askhat Myngbay , Sergey Yegorov , Baurzhan Negmetzhanov, Irina Kadyrova, Angelina Yershova, Ulpan Kart , Matthew S. Miller and Gonzalo Hortelano

Supplementary Materials:

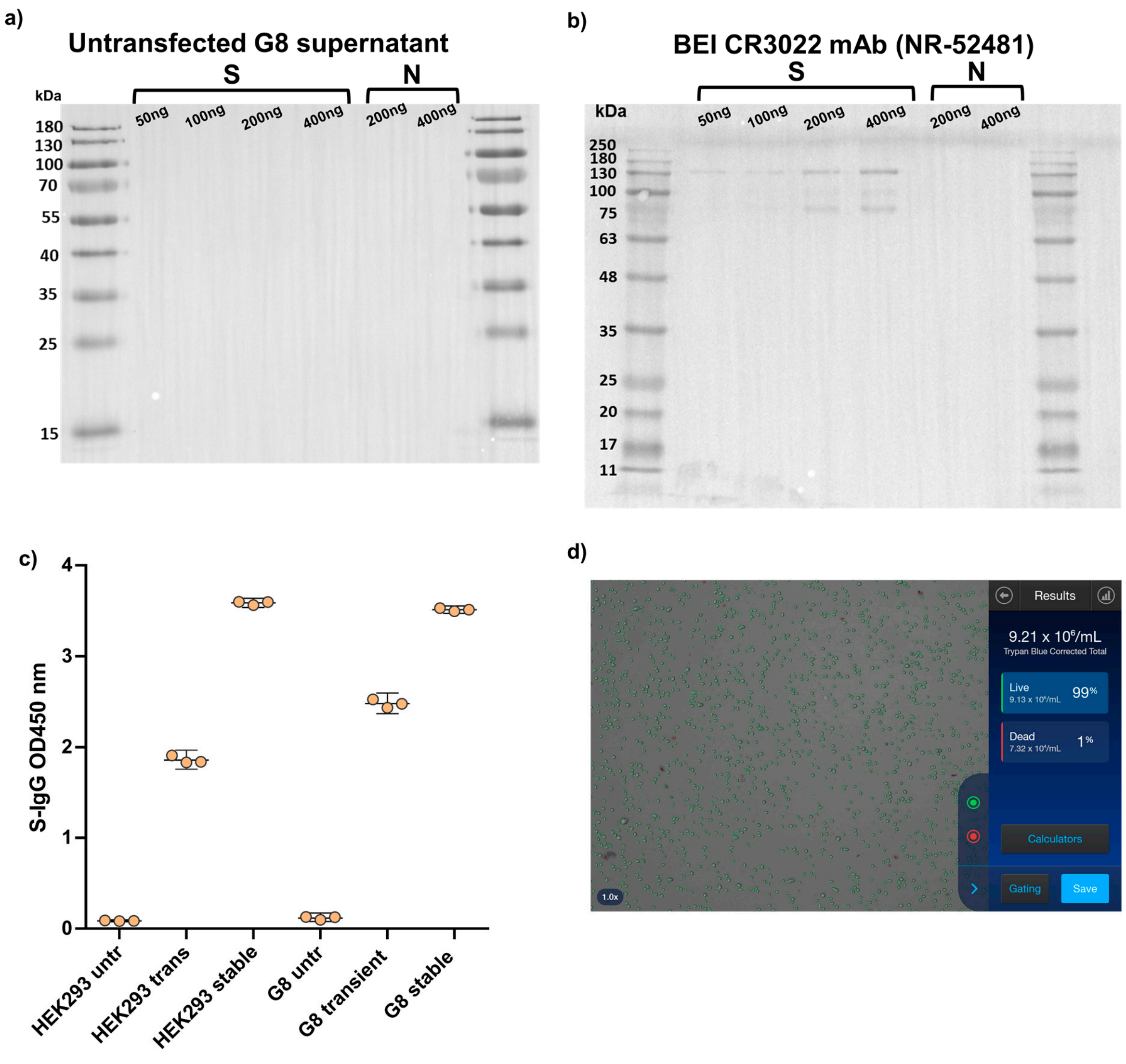

Figure S1. SARS-CoV-2 antibody detection by Western blotting and ELISA. a,b) Western blotting of supernatants from control (un-transfected) G-8 cells (a) and diluted

BEI CR3022 mAb (b); different amounts of S (50-400 ng) and nucleocapsid (N, 200-400 ng) proteins were separated on a 10% SDS–PAGE gel, transferred onto a PVDF membrane and then blotted with G-8 cell culture supernatant; probing in a, b was done using a HRP-conjugated rabbit IgG specific to both the heavy and light chains of human IgG. Lanes M: protein ladder; Lanes S: S protein at 50, 100, 200 and 400 ng. Lanes N: N protein at 200 and 400 ng. c) Representative ELISA plot of S-IgG binding for HEK293 and G-8 cell supernatants. Cell supernatants were incubated on plates coated with S and RBD proteins. Supernatants were collected at 48h post-encapsulation. Lines and brackets represent the geometric means and 95% confidence intervals, respectively. OD, optic density. d) Representative output from the automated cell counter showing the count and viability of cells retrieved from microcapsules.

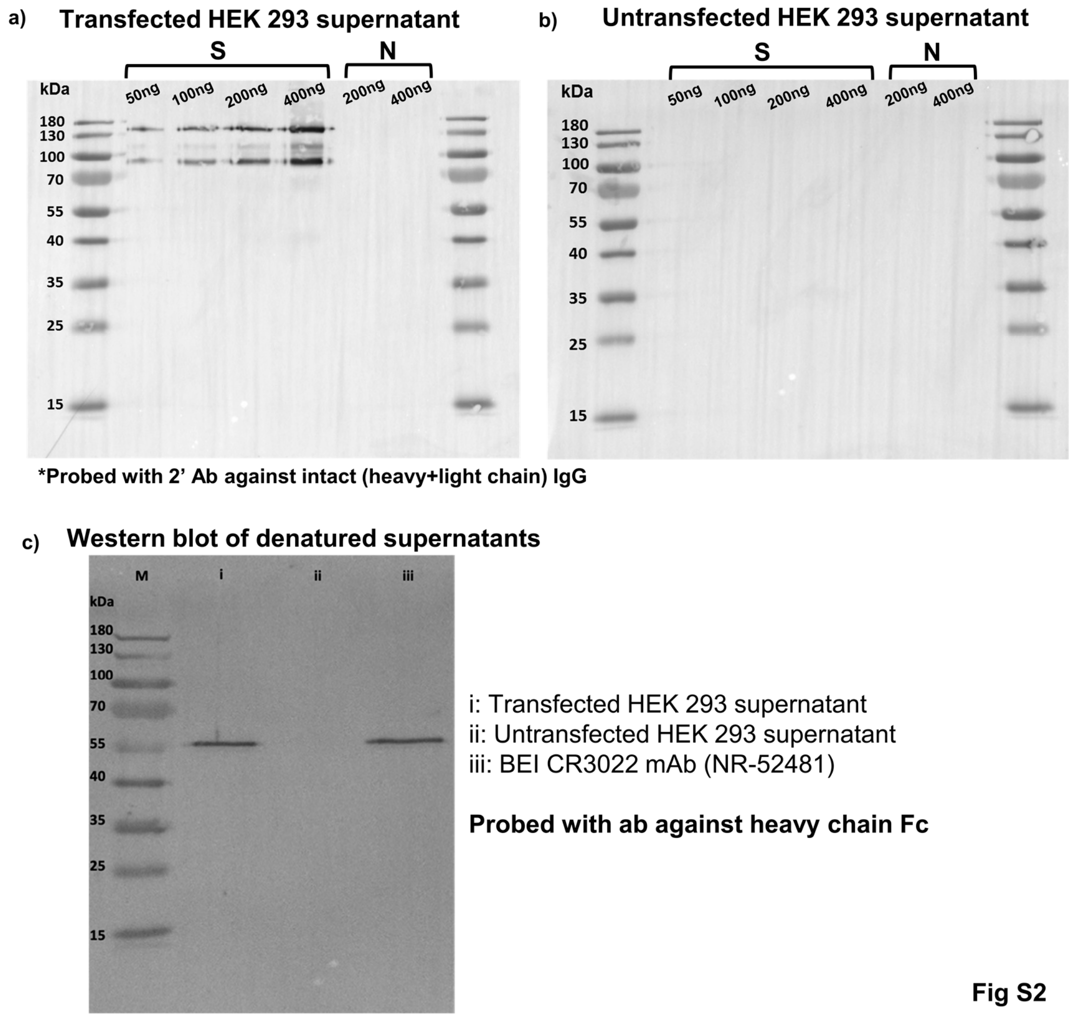

Fig S2

Figure S2. SARS-CoV-2 monoclonal antibody production in cells transfected with the CR3022 heavy and light chains. In panels a,b different amounts of S (50-400 ng) and nucleocapsid (N, 200-400 ng) proteins were separated on a 10% SDS–PAGE gel, transferred onto a PVDF membrane and then blotted with HEK-293 cell culture supernatant; probing was done using a HRP-conjugated rabbit IgG specific to both the heavy and light chains of human IgG. Lanes M: protein ladder; Lanes S: S protein at 50, 100, 200 and 400 ng. Lanes N: N protein at 200 and 400 ng. In panel c, purified cell culture supernatants and the BEI CR3022 mAb were denatured prior to Western blotting; probing was done using a HRP-conjugated goat IgG specific to the heavy chain Fc region of human IgG. kDA=kilodaltons.

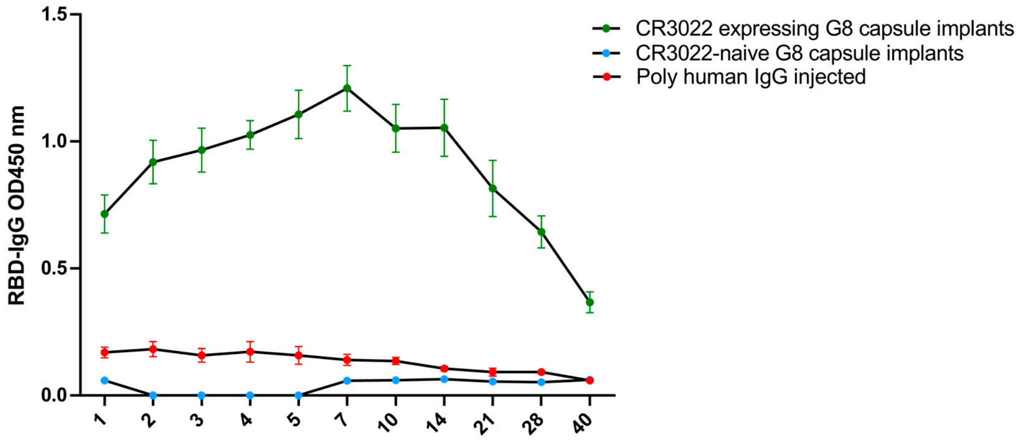

Figure S3. SARS-CoV-2 monoclonal antibody detection in mice over 40 days of implantation.

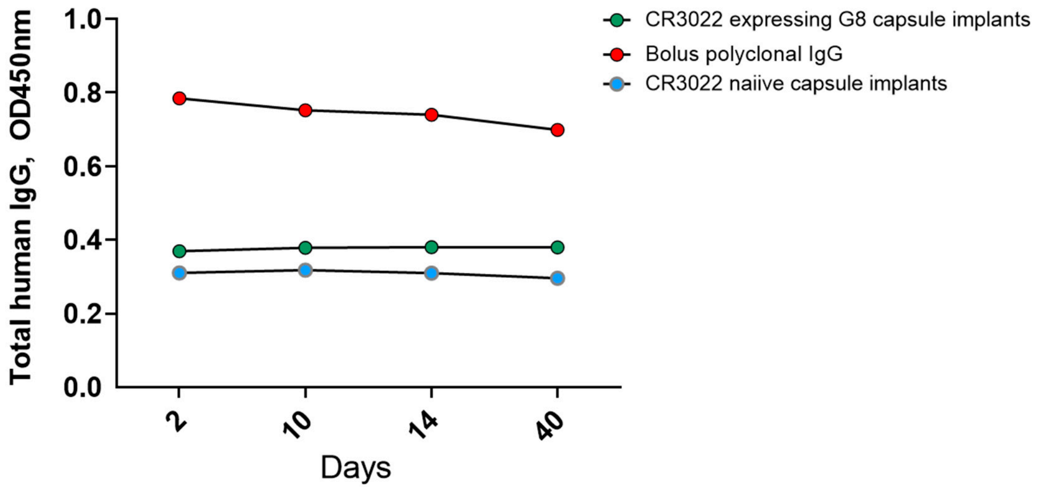

Figure S4. Total human IgG detection in mice over 40 days of implantation.
